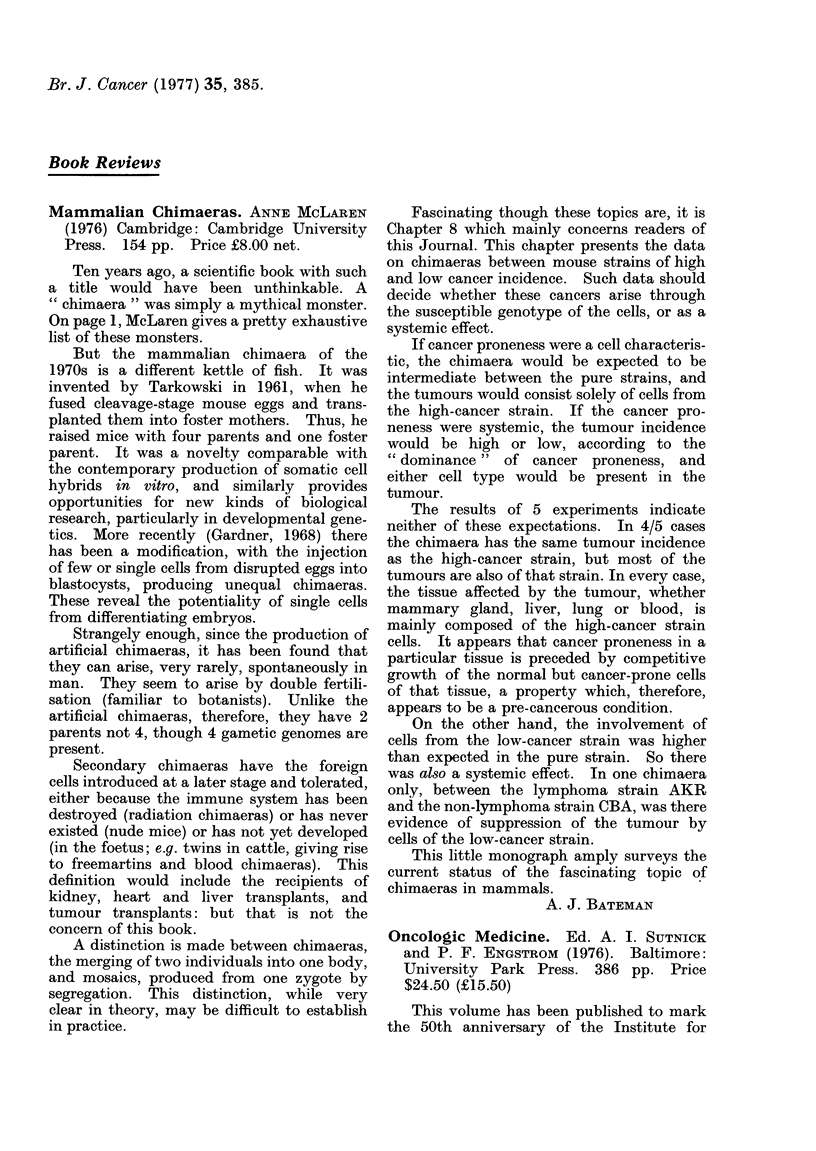# Mammalian Chimaeras

**Published:** 1977-03

**Authors:** A. J. Bateman


*Br. J. Cancer* (1977) **35**, 385.

### *Book Reviews*

**Mammalian Chimaeras.** ANNE MCLAREN (1976) Cambridge: Cambridge University Press. 154 pp. Price £8.00 net.

Ten years ago, a scientific book with such a title would have been unthinkable. A " chimaera " was simply a mythical monster. On page 1, McLaren gives a pretty exhaustive list of these monsters.

But the mammalian chimaera of the 1970s is a different kettle of fish. It was invented by Tarkowski in 1961, when he fused cleavage-stage mouse eggs and transplanted them into foster mothers. Thus, he raised mice with four parents and one foster parent. It was a novelty comparable with the contemporary production of somatic cell hybrids *in vitro*, and similarly provides opportunities for new kinds of biological research, particularly in developmental genetics. More recently (Gardner, 1968) there has been a modification, with the injection of few or single cells from disrupted eggs into blastocysts, producing unequal chimaeras. These reveal the potentiality of single cells from differentiating embryos.

Strangely enough, since the production of artificial chimaeras, it has been found that they can arise, very rarely, spontaneously in man. They seem to arise by double fertilisation (familiar to botanists). Unlike the artificial chimaeras, therefore, they have 2 parents not 4, though 4 gametic genomes are present.

Secondary chimaeras have the foreign cells introduced at a later stage and tolerated, either because the immune system has been destroyed (radiation chimaeras) or has never existed (nude mice) or has not yet developed (in the foetus; *e.g.* twins in cattle, giving rise to freemartins and blood chimaeras). This definition would include the recipients of kidney, heart and liver transplants, and tumour transplants: but that is not the concern of this book.

A distinction is made between chimaeras, the merging of two individuals into one body, and mosaics, produced from one zygote by segregation. This distinction, while very clear in theory, may be difficult to establish in practice.

Fascinating though these topics are, it is Chapter 8 which mainly concerns readers of this Journal. This chapter presents the data on chimaeras between mouse strains of high and low cancer incidence. Such data should decide whether these cancers arise through the susceptible genotype of the cells, or as a systemic effect.

If cancer proneness were a cell characteristic, the chimaera would be expected to be intermediate between the pure strains, and the tumours would consist solely of cells from the high-cancer strain. If the cancer proneness were systemic, the tumour incidence would be high or low, according to the " dominance " of cancer proneness, and either cell type would be present in the tumour.

The results of 5 experiments indicate neither of these expectations. In 4/5 cases the chimaera has the same tumour incidence as the high-cancer strain, but most of the tumours are also of that strain. In every case, the tissue affected by the tumour, whether mammary gland, liver, lung or blood, is mainly composed of the high-cancer strain cells. It appears that cancer proneness in a particular tissue is preceded by competitive growth of the normal but cancer-prone cells of that tissue, a property which, therefore, appears to be a pre-cancerous condition.

On the other hand, the involvement of cells from the low-cancer strain was higher than expected in the pure strain. So there was *also* a systemic effect. In one chimaera only, between the lymphoma strain AKR and the non-lymphoma strain CBA, was there evidence of suppression of the tumour by cells of the low-cancer strain.

This little monograph amply surveys the current status of the fascinating topic of chimaeras in mammals.

A. J. BATEMAN